# Frailty after Liver Transplantation: A Complex Unexplored Issue

**DOI:** 10.3390/jcm13154537

**Published:** 2024-08-02

**Authors:** Filippo Gabrielli, Filippo Biagi, Alessandra Avossa, Margherita Falcini, Fabio Nascimbeni, Pietro Andreone, Stefano Gitto

**Affiliations:** 1 Internal and Metabolic Medicine, Department of Medical and Surgical Sciences for Children & Adults, AOU of Modena, University of Modena and Reggio Emilia, 41126 Modena, Italy; filippo.gabrielli3@gmail.com (F.G.);; 2 Department of Surgical Sciences, University of Bologna, 40126 Bologna, Italy; 3 Internal Medicine, University Hospital Careggi and Department of Experimental and Clinical Medicine, University of Florence, 50134 Florence, Italy; 4 Postgraduate School of Allergology and Clinical Immunology, University of Modena and Reggio Emilia, 41126 Modena, Italy

**Keywords:** frailty, liver transplantation, sarcopenia, frailty assessment tools

## Abstract

Frailty is a multidimensional syndrome predominantly studied in the elderly, characterized by reduced resistance to stressors due to diminished physiological reserve and resilience. Advances in surgical techniques and immunosuppressive drugs have improved long-term survival rates in solid organ transplant recipients, yet the 10-year survival is satisfying. However, liver transplant recipients have a noteworthy risk of developing frailty status. After liver transplant, frailty can be favored by socioeconomic, cultural, and health-related factors, leading to increased risks of hospitalization, morbidity, and mortality. Various tools for frailty assessment exist, but none are universally validated for post-transplant patients. The integration of socioeconomic and psychological factors into frailty evaluation could improve quality of life and long-term outcomes for transplant recipients. Multidisciplinary approaches, including psychosocial support, are essential for managing frailty and enhancing the overall care of transplanted patients. This narrative review aims to comprehensively address the principal frailty risk factors associated with liver transplantation.

## 1. Introduction

### 1.1. Frailty and Its Definitions

Frailty is a multidimensional concept that has primarily been described in the field of geriatric medicine. It is defined as a state of reduced resistance to external stressors due to diminished physiological reserves and resilience [1]. It was the research group led by Fried et al. in 2001 that described it as a syndrome associated with weight loss, exhaustion, weakness, slowed walking speed, and reduced physical activity [2]. This syndrome is influenced by several predisposing factors, including socioeconomic and cultural factors, as well as health-related factors such as the number of chronic diseases and comorbidities. It is associated with an increased incidence of accidental falls, a rise in dependency, a decrease in quality of life (QOL), and a heightened risk of hospitalization and mortality [2]. In 2012, international consensus expanded the definition of frailty to encompass not only physical aspects but also psychological vulnerability [3]. Therefore, frailty should be understood as a multidomain syndrome that affects not only the physical dimension but also cognitive, emotional, and psychosocial aspects, leading to a worsened prognosis in affected individuals [2,4].

Frailty presents some critical characteristics: Firstly, it is dynamic, meaning that the same individual may, and often does, fluctuate between different levels of frailty. Secondly, it is potentially preventable and, to some extent, treatable [5]. Thus, it appears crucial to be able to assess, quantify, and treat it via a standardized approach.

### 1.2. Liver Transplant

Although, in recent decades, there has been a significant improvement in surgical techniques and immunosuppressive drugs, which has led to the enhanced long-term survival of patients with solid organ transplants [6], for orthotopic liver transplantation (OLT), the 10-year survival rate has remained at 61–65% [7]. Additionally, in recent decades, the eligibility criteria for OLT and the prevalence of etiologies of chronic liver disease leading to OLT have also changed. Specifically, the age of recipients has increased and the contribution of viral hepatitis has decreased, making metabolic-associated fatty liver disease (MAFLD) the second most common indication for OLT, following alcoholic liver disease [8,9,10]. The European guidelines for OLT, although not formally setting an age limit for transplantation, recommend a multidisciplinary evaluation for recipients over 65 years old. They emphasize that recipients over 70 years old are at a higher cardiovascular risk compared to younger recipients [8,11,12]. According to US estimates, in 2021, approximately 22.4% of OLT patients were over 65 years old, while in Europe, 19.4% of liver recipients were over 65 years old [6,13]. Therefore, multidisciplinary teams responsible for evaluating whether to place a patient on the waiting list must assess older patients with complex medical and socioeconomic conditions. A necessary evaluation for this type of patient, which can predict post-OLT outcomes, is the measurement of frailty [14].

### 1.3. Frailty in Cirrhosis—Liver Frailty Index

Unlike in the field of geriatrics, from which the concept derives, in hepatology, frailty mostly refers to functional impairment and physical frailty, both of which are strictly related to sarcopenia, a well-known complication of cirrhosis [4]. 

Frailty is a common condition in patients with advanced liver disease. A prevalence of frailty ranging from 18% to 43% has been estimated in patients with advanced liver disease. However, there is variability in prevalence depending on the severity of the disease, comorbidities, and measurement tools [4]. As liver function deteriorates, a cascade of processes ensues, involving muscle catabolism, [15], increased energy expenditure [16], and alterations in gluconeogenesis and fatty acid oxidation [17], resulting in malnutrition and sarcopenia, thereby reducing QOL [18,19]. The pathophysiological pathways leading to frailty and the liver-specific determinants in the setting of chronic liver diseases go beyond the scope of this review and, thus, will not be covered [4]. Therefore, a patient on the waiting list for an OLT may present with compound sarcopenia, characterized by progressive muscle loss associated with aging and a component attributable to chronic liver disease [20,21]. Moreover, in some individuals, obesity sarcopenia cannot be excluded. 

The concept of frailty is clinically relevant in the field of hepatology and particularly in the pre-transplant phase, as it has been found to be an independent predictor of mortality or cirrhosis progression, unplanned hospitalization in patients affected by liver cirrhosis, pre- and post- OLT morbidity and mortality, and waitlist mortality in transplant candidates [22,23,24,25,26,27].

Recent studies have demonstrated that compared to non-frail patients, frail candidates experienced longer hospital stays, shorter one-year survival rates, and an increased risk of early post-transplant complications [28,29]. Interestingly, as Xu et al. reported, it appears that a frail phenotype is more frequently found in specific etiologies of cirrhosis; nonetheless, they reported that, regardless of the etiology, frailty was associated with waitlist mortality [30]. Various tools have been proposed to assess sarcopenia and/or frailty in patients undergoing OLT, including the skeletal muscle index (SMI), activities of daily living, Karnofsky performance status (KPS), the clinical frailty scale, the Fried frailty phenotype [2], the gait speed test, the 6-minute walking test, cardiopulmonary exercise testing, and the short physical performance battery [31]. The Fried frailty test is commonly used for assessing frailty, but it has demonstrated limitations in cirrhotic patients because the domains evaluated by the test can be affected by disease complications [23,26]. In 2017, Lai et al. introduced a novel index, the Liver Frailty Index (LFI), to better characterize the different phenotypes of patients with chronic liver diseases and overcome the limitations of the already existing scores, namely the MELD-Na, which did not directly include extrahepatic complications of cirrhosis such as sarcopenia, malnutrition, encephalopathy, and functional decline [32]. The idea behind the LFI was to improve risk stratification and risk prediction of waitlist mortality [32]. The test includes the evaluation of chair stands, balance testing, and grip strength. Although Singh et al. reported no difference in frailty assessment among cirrhotic patients using the Fried frailty phenotype, the clinical frailty scale, and the short physical performance battery, the FLI test is the most validated test in cirrhotic patient cohorts [33,34]. Although a valid tool for frailty assessment, the LFI only evaluates physical frailty, and it lacks validation for hospitalized patients; thus, it should not be used as the sole parameter for assessing a patient. 

### 1.4. Frailty in Liver Transplant Recipients

With recent advances in the field of transplantation and the increase in life expectancy, it is going to be more and more common to treat complex patients, affected by multiple comorbidities and a frail phenotype. Given the clinical, social, and economic burden that frailty is expected to cause in this specific population, the lack of data on this topic is concerning. On one hand, there is a fair amount of evidence regarding frailty before OLT; on the other hand, data are scarce on frailty evolution, assessment, and management after-transplant, which remains, as of today, a largely unexplored issue. A big limitation in handling this complex issue is that most of the literature regarding frailty in OLT has predominantly focused on functional impairment and sarcopenia, leaving the psychological side of it less investigated, partly due to the presence of possible confounding factors (i.e., encephalopathy) [35]. 

Objective assessment of physical impairment can be achieved by the application of a number of scales and tests, most of which have been validated in specific settings but that, as of today, lack validation in the setting of OLT; thus, despite multiple tools being available, it is not yet clear which tools perform best in this specific setting [18]. On the other hand, the assessment of sarcopenia, although requiring specific machinery and associated to a certain degree of invasiveness, is more standardized. In an attempt to standardize and ease frailty evaluation in clinical practice, in a 2023 review, Tandon et al. suggested combining an objective screening test with imaging to identify sarcopenia to provide a frailty assessment in the outpatient OLT population [35]. Furthermore, the American Society of Transplantation has proposed the assessment of frailty in the setting of OLT through a tool kit comprising the KPS scale, ADL/IADLs, the Liver Frailty Index, and the 6-minute walk test [36]. The above-mentioned scales and indexes can be used for the pre-OLT assessment of frailty while post-OLT tools are not available.

### 1.5. Scope and Definition

Frailty in OLT patients is a critical issue that can worsen the prognosis of affected patients both in the immediate post-OLT period and in the long term. Its multidomain origin and dynamic nature can make its identification and monitoring challenging. Therefore, our aim was to define the elements that can contribute to post-transplant frailty and its monitoring.

## 2. Methods

The current literature was reviewed to assess the state of the art of frailty assessment after OLT and its clinical implications. We carried out a non-systematic search on PubMed, MEDLINE, Google Scholar, Ovid, Scopus, and Web of Science using the following search words: “morbidity”, “mortality”, “frailty”, “hospitalization”, and “quality of life”; these were all combined with “liver transplant”. We considered all papers reporting human-related data (inclusion criteria), excluding articles with unavailable full text, articles not written in the English language, and abstracts, book chapters, and articles published before 1990 (exclusion criteria). 

## 3. Potential Predictors of Frailty after Liver Transplant

As of today, there is no universally accepted and validated tool to assess frailty after OLT, and for that matter after any solid organ transplantation. The importance of post-transplant frailty evaluation lies in the fact that not all contributors are reversible with the implantation of the donor organ; additionally, the procedure itself is a potential contributor to the worsening of the patient’s physiological reserve as it is burdened by a significant risk of complications in up to half of the patients [37]. 

In a 2018 study, Lai et al. examined a cohort of OLT recipients to assess the post-transplant evolution of frailty using the LFI; their results showed an improvement of robustness at 12 months after a temporary decline at 3 months [38]. The same group of researchers found from data derived from eight US centers that frail OLT recipients, as defined by a LFI ≥ 4.5, had a higher risk of death and heavier health resource utilization compared to the non-frail counterpart [39]. Notably, pre-transplant functional status predicts postoperative morbidity and mortality after OLT [40]. In particular, physical frailty assessed by the short physical performance battery offers relevant prognostic details for cirrhotic patients undergoing LT.

Another study conducted by Raveh et al. aimed to provide a score, the Frailty Severity Index (FSI), to predict various OLT outcomes, such as one-year survival, a stay longer than six days in an intensive care unit, major complications within six months post-transplant, and the need for mechanical ventilation beyond 24 h [41]. The FSI takes into account three domains: physical performance through the KPS and the National Pressure Ulcer Advisor Panel Scale [42], nutrition assessment through the Modified Academy/Aspen [43] measure, and the severity of liver disease and inflammation through the evaluation of serum albumin, cholesterol, and lymphocytes. The score has been shown to significantly predict one-year survival in OLT patients, as well as the development of major complications within one year [41]. However, this study exhibited numerous limitations, including its retrospective nature, the absence of internal validation, intra-observer and inter-observer variability, and a lack of simplicity in calculating the involved variables, making it difficult to apply in the outpatient setting. As previously mentioned, one of the factors influencing frailty is sarcopenia, which can be supported by protein–calorie malnutrition, reduced physical activity, and frequent rapid exhaustion in patients on the waiting list for OLT [44]. Nutrition plays a crucial role in OLT recipients, as there is an increased need for protein and calories during the first 4 weeks post-transplantation [45]. A nutritional assessment should be performed in post-OLT patients, including a high-protein diet to slow the progression of sarcopenia [46]. Furthermore, the use of immunomodulatory drugs, an unhealthy lifestyle, and psychosocial issues such as depression or social isolation can lead OLT recipients to experience a deterioration in their performance status, exposing them to reduced survival [47,48,49].

The challenges in finding a solid score to predict patient’s frailty in the post-transplant setting lie in the multiple determinants of this multidimensional condition. 

Indeed, the domains of potential interference on frailty in an OLT patient can encompass both medical factors, including the liver disease that led to transplantation and immunotherapy, as well as social, psychological, and lifestyle-related factors [50,51,52].

## 4. Metabolic Disorders

In a recent analysis, conducted on an Italian cohort of very-long-term survivors after OLT, it emerged that after surpassing the first period burdened by liver-related mortality, long-term mortality is associated with increased risk of metabolic, cardiovascular, and oncologic comorbidities [53]. The metabolic syndrome and its component factors, including arterial hypertension, insulin resistance, dyslipidaemia, and central obesity, are prevalent among OLT recipients [54]. The high prevalence of these pathologies in OLT recipients has several causes. Regarding the pre-transplant period, in recent decades, there has been a progressive increase in cases of cirrhosis in patients with MAFLD, a liver disease triggered by the same factors as metabolic syndrome. This has made MAFLD the second leading cause of OLT in the USA [6]. Furthermore, similar to the general population, there has been an increase in the prevalence of type 2 diabetes mellitus (T2DM) and overweight/obesity among patients eligible for transplantation [55]. Regarding the post-OLT period, patients may experience the onset of de novo occurrence of these pathologies or their exacerbation due to immunosuppressive therapy [48] or an unhealthy lifestyle [56,57,58]. A recent meta-analysis has highlighted that patients with metabolic syndrome exhibit an increased risk of frailty (OR 1.73, 95% CI, 1.41–2.13) [59]. Therefore, metabolic disorders in transplant patients can lead to frailty and should be taken into account in the assessment of post-OLT patients

### 4.1. Cardiovascular Risk

The transplant recipient carries a high risk for cardiovascular diseases, as suggested by the guidelines of the European Society of Cardiology [60]. Cardiovascular diseases are the leading cause of mortality in the first year and the third most common cause after one year following OLT [61,62,63]. As mentioned earlier for metabolic disorders, the post-OLT patient presents cardiovascular risk factors inherited from pre-OLT conditions that can be exacerbated by immunosuppressive therapy or an unhealthy lifestyle. Furthermore, cardiovascular risk and frailty are interconnected. They recognize common risk factors, and both can reinforce each other: lifestyle-related factors such as lack of physical exercise, poor diet, socioeconomic stressors, advanced biological aging, non-cardiovascular diseases, and neoplasms can increase both cardiovascular risk and frailty [64]. Although the correlation between cardiovascular risk and frailty has been explored in the general population, the literature is lacking in the assessment of this issue in OLT patients.

### 4.2. Oncological Risk

De novo malignancies represent a significant concern in OLT patients. The risk of developing an oncological disease in OLT patients is attributed not only to immunosuppressive therapy but also to factors such as aging, lifestyle, and chronic infections [65]. OLT patients have an 11-fold higher risk than the general population of developing de novo neoplasms, and these neoplasms often exhibit more aggressive behaviors and are less responsive to therapies [66]. In addition, for the same type of tumor, survival is worse in OLT patients compared to the general population [67]. Immunosuppression plays a significant role not only through the reduction in immunosurveillance but also due to the development of insulin resistance [68] and the direct carcinogenic effects of certain immunosuppressants [69]. Frailty in the oncological field is a well-studied concept as it represents a limitation to therapeutic success, particularly in the elderly [70]. In this field, the domains assessed also include social, physical, nutritional, and cognitive, and they are dependent on the number of comorbidities and medications the patient is taking [70]. Frailty in oncology patients is associated with an increased risk of chemotherapy intolerance and postoperative complications [71,72].

### 4.3. Quality of Life and Psychosocial Elements

The World Health Organization (WHO) defines quality of life as an individual’s perception of their position in life within the cultural and value contexts in which they live, in relation to their goals, expectations, standards, and concerns [73]. Multiple reports highlight the unsurprising finding that OLT carries an improvement in QOL; nonetheless, it remains unclear whether it reaches a level similar to that of the general population [74,75]. One of the key determinants of QOL is physical activity, on which there is a vast and well-consolidated body of evidence. Regular physical activity provides protection against chronic diseases, frailty, disability, falls and mortality [76,77], whereas its reduction is a known risk factor for obesity, T2DM, cardiovascular diseases, and death [78,79]. Recently, we reported data from a large multicenter study showing an inadequate level of physical activity among clinically stable OLT patients and its impact on QOL [56].

Furthermore, in recent years, there has been growing interest in the concept of “prehabilitation” in the surgical field [80]. The term “prehabilitation” refers to programs of physical activity, supervised home exercises, and educational interventions on nutrition and lifestyle that have demonstrated a reduction in procedure costs before the surgical procedure and shown an improvement in surgical outcomes [80]. Prehabilitation interventions in the context of liver transplantation have shown a reduction in the progression of sarcopenia and, in some cases, even a reversal of the condition [80,81,82,83,84,85,86,87]. 

An early rehabilitation after transplant is mandatory to limit the risk of metabolic and cardiovascular impairment. Today, to the best of our knowledge, specific rehabilitation programs are not available. Further studies should propose personalized rehabilitation programs for transplant recipients that show, as we widely demonstrated, many specific points of weakness.

Other important factors to consider in OLT patients are socioeconomic status and health literacy levels: indeed, a low socioeconomic status has been correlated with an increased risk of death at 2 years post-transplantation, and reduced health literacy is inherently linked to lower treatment adherence, thereby increasing the risk of mortality [88,89].

Last but not least, patients entering the OLT process require psychological and psychiatric assessment. Psychological/psychiatric factors that can impact treatment effectiveness can be identified in all phases of the process. Particularly in the post-OLT phase, patients may resume alcohol consumption, experience depression, or feel isolated [51,90]. If these factors are identified early and treated appropriately, post-OLT survival improves [91,92,93]. 

Specifically, according to a meta-analysis, depression is associated with a 65% increase in the risk of post-transplant mortality [94]. The key factor that could improve frailty and quality of life in post-OLT patients might be physical activity. In fact, it has been found that about one-quarter of OLT patients engage in low levels of physical activity or do not engage in it at all [56]. In the geriatric field, good results in improving social frailty have been observed with interventions based on social-resource domains, self-management, and social behavior [95]. These interventions have demonstrated a significant impact on the sensation of loneliness [96] but remain confined to the geriatric field. Therefore, further studies need to be conducted on OLT patients with frailty.

The Short Form Health Survey (SF-12) is a 12-item questionnaire that measures eight domains of physical and psychological health. It is feasible and repeatable in an outpatient setting for assessing quality of life and also used in patients undergoing OLT [97,98]. 

An important psychological component that should be explored in patients before and after OLT is resilience. Resilience refers to the individual’s capacity to persevere despite adversity [99]. Resilience involves three fundamental elements: significant adversity or risk, the availability of resources or tools to mitigate the effects of the adversity, and a positive response or the prevention of negative outcomes [100]. A study conducted by Lai et al. demonstrated that low resilience was associated with frailty in patients with liver cirrhosis [101].

### 4.4. Immunosuppressive Therapy

Immunosuppressive therapy is a cornerstone of long-term success in liver transplantation; however, it is associated with significant side effects, including increased risk of hypertension, diabetes mellitus, hyperuricemia, weight gain, and dyslipidemia and the development of neoplasms. Therefore, the prevention of these conditions, along with the individualization of immunosuppressive therapy, is essential in the management of liver transplant patients [102]. Frailty is an important variable in the management of immunosuppressive therapy. Large-scale studies are needed to develop a score that can grade the severity of frailty in liver transplant patients, especially for optimizing therapeutic management [103]. Sarcopenia, increased fat mass, and loss of lean body mass cause variations in the pharmacokinetics of immunosuppressants, potentially reducing their efficacy and increasing side effects [104]. For example, the reduction in lean body mass and the increase in adipose tissue result in a lower volume of distribution. In obese patients, calcineurin inhibitors have prolonged half-lives due to their binding with circulating lipoproteins [103,105]. It should also be noted that frail patients may be exposed to a greater number of medications for other conditions [106]. The concurrent use of immunosuppressive drugs can lead to drug interactions or adverse drug events, potentially worsening the psychophysical performance of the frail patient [106].

## 5. Conclusions

**Figure 1:**
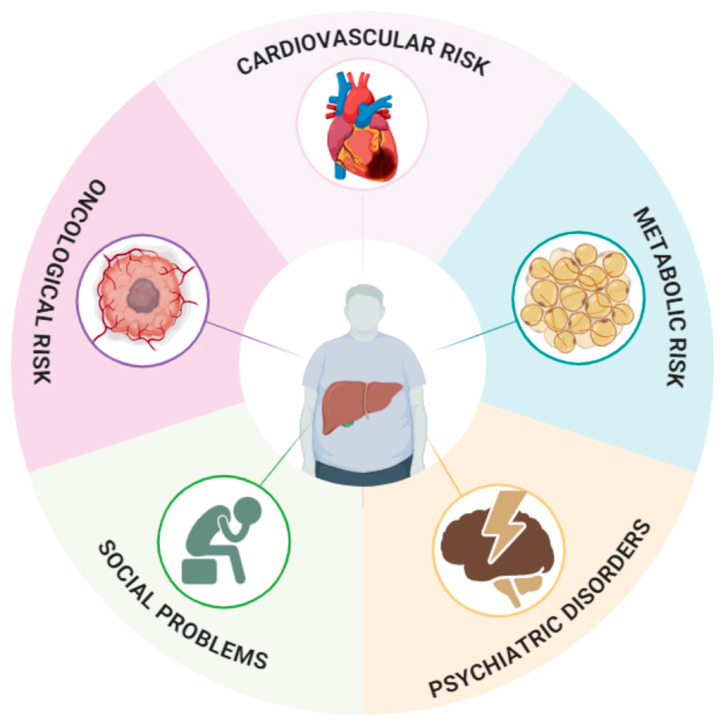
The main factors that can potentially favor the development of frailty status in liver transplant recipients. Psychiatric disorders and social problems, both pre-existing and de novo post-transplant, such as depression, loneliness, and relapse into alcohol use, as well as immunosuppressive therapy—which may increase the risk of de novo malignancies—and a sedentary lifestyle that contributes to cardiovascular risk, are factors that can contribute to frailty in post-OLT patients.

As summarized in Figure 1, many different factors can enhance frailty status after OLT. Transplanted patients are at high risk of being physically frail because of their condition or age, and this vicious cycle can be supported by emotional, social, and economic frailties that can hardly be evaluated by a single score.

Much like in geriatric medicine, in the transplant field, there is a need for a shift from a disease-oriented to a function-oriented approach: social, clinical, and biologic characteristics of older adults do not reflect those on which international recommendations and guidelines are developed.

The lack of assessment tools and the need for their validation in this specific population should be a focus for future research. These studies will also need to consider the dynamic nature of frailty. In the future, based on more precise and targeted studies of this population, it will be possible to develop guidelines and protocols for managing frailty in OLT patients. 

Inadequate nutritional status in OLT patients plays a critical role in the development and persistence of frailty. Therefore, in addition to anthropometric measurements, prealbumin, and the Subjective Global Assessment (SGA), the Royal Free Hospital Nutritional Prioritizing tool could be considered. Although not specifically designed for post-OLT patients, it has demonstrated potential as an assessment tool [107].

Finally, it is important to emphasize that the tools designed to assess frailty in liver transplant patients should prioritize clinical accessibility and reproducibility, especially in an outpatient setting. At present, there is no existing score that evaluates frailty in liver transplant patients while accounting for other post-transplant variables such as immunosuppressive therapy and polypharmacy.

The main limitation of the present study is that it does not have a systematic approach. Despite this, this is one of the first attempts to analyze the issue of frailty as an element characterizing the post-transplant period.

In conclusion, transplanted patients require logistical, social, clinical, nutritional, and psychological support throughout their lives. In this sense, encouraging social aggregation, for example, through associations and volunteering, could be a further significant element of support.
